# Phase Transitions and Hysteresis for a Simple Model Liquid Crystal by Replica-Exchange Monte Carlo Simulations

**DOI:** 10.3390/molecules26051421

**Published:** 2021-03-05

**Authors:** Akie Kowaguchi, Paul E. Brumby, Kenji Yasuoka

**Affiliations:** Department of Mechanical Engineering, Keio University, 3-14-1 Hiyoshi, Kohoku-ku, Yokohama 223-8522, Japan; akie.kowaguchi@keio.jp

**Keywords:** Monte Carlo, replica exchange, liquid crystal

## Abstract

In this work, the advantages of applying the temperature and pressure replica-exchange method to investigate the phase transitions and the hysteresis for liquid-crystal fluids were demonstrated. In applying this method to the commonly used Hess–Su liquid-crystal model, heat capacity peaks and points of phase co-existence were observed. The absence of a smectic phase at higher densities and a narrow range of the nematic phase were reported. The identity of the crystalline phase of this system was found to a hexagonal close-packed solid. Since the nematic-solid phase transition is strongly first order, care must be taken when using this model not to inadvertently simulate meta-stable nematic states at higher densities. In further analysis, the Weighted Histogram Analysis Method was applied to verify the precise locations of the phase transition points.

## 1. Introduction

Despite the significant impact that liquid crystals have had in the development of portable display devices, there remains much that is incomplete in our understanding of their complex phase behavior. The precise prediction of phase transition points is a challenging task when using molecular simulations. This is due to the fact that simulation trajectories may often become trapped in local minima rather than their most stable phase [1].

When simulating liquid crystals, it is desirable to be able to capture the essential molecular properties that give rise to the multitude of different phases that are observed experimentally. The most common of these are the isotropic, nematic, and smectic phases. The isotropic phase is characterised by both random positioning and orientation. With decreasing temperature and increasing pressure, molecules gain orientational order [2]. This is the so-called nematic phase. At yet higher densities, the smectic phases result in layered structures which also possess some positional ordering [3].

There are several well-established molecular models which have seen widespread use in simulation studies of liquid crystals. For example, in molecular dynamics simulations of systems of particles interacting via the Gay–Berne potential, all of the aforementioned phases may be observed [4]. With the hard spherocylinder model, Frenkel discovered that the system can exist in both nematic and smectic phases [5]. Hard ellipsoids, on the other hand, cannot form stable smectic phases [6]. The latter point aptly demonstrates the importance of selecting an appropriate molecular model if one wishes to study the phase behaviour of a specific liquid crystal.

In addition to the previously discussed liquid-crystal molecular models, the Hess–Su model is a more recent development [7]. It represents the interactions between slightly elongated molecules and has some unique features that make it particularly attractive for both simulators and theoreticians. The model has a pair interaction potential that is a modification of the standard Lennard–Jones model. The attractive term has an additional anisotropic contribution from the intermolecular pair interaction, which varies according to the relative positions and orientations of the molecules. With the Hess–Su model, the computational costs are generally lower than they are with other models and it is relatively straightforward to implement in both simulations and theories [8,9]. In simulations, this model exhibits isotropic and nematic liquid-crystal phases as well as crystalline solids, but the phase transition points are, as yet, not precisely known.

Greschek and Schoen [10] looked at surface prewetting for various orientationally dependent surface interaction types, each of which resulted in different molecule surface-anchoring behaviour. Their main findings were that the enhancement of orientational ordering at the surface leads to surface wetting with the formation of thick surface layers. In similar work with slit-pore systems, it was found that variations in surface interactions may cause shifts in the phase transition points [11]. Similarly, Melle et al. [12] looked at the surface interactions around spherical patchy colloids and found novel defect topologies. In a closely related study, Steiger et al. [13] performed a similar analysis with Janus colloids.

For liquid-crystal phases, which exhibit wide variation in their positional and orientational properties, there are often significant density differences at the phase transition points. This difference is often the cause of hysteresis in simulation results. For example, hysteresis has been observed for many commonly used molecular models, including Gay–Berne [14,15], hard ellipsoids [6], spin crossover materials [16], and square-well and soft-repulsive spherocylinders [17].

In the Monte Carlo (MC) simulation studies of Steuer et al. [18,19], strong hysteresis at the nematic–isotropic and nematic–solid phase transitions were observed in both approaches of incrementally increasing and decreasing the pressure. The extent of this hysteresis was particularly noticeable between the nematic and solid phases, with transitions found to occur at reduced pressures of 7.4 from the nematic to solid phases, while the opposite phase transition from solid to nematic required a pressure as low as 1.5 [19]. This pressure is actually low enough to subsequently transition to an isotropic phase, effectively bypassing the nematic phase. The authors considered that this large discrepancy was due to the strongly first-order nematic–solid phase transition, as well as finite size effects and practical simulation time limitations. It is worth noting that the error bars in the work of Steuer et al. are greater than would be expected for equilibrium systems with N=1000 particles. This leads us to believe that the presented results may not have been run for a sufficient number of MC cycles. In their work, simulations were limited by the computers available at the time and were performed over 10,000 MC cycles.

Fortunately, there are methods available to efficiently sample the low-energy (high density) regions in molecular simulations. One example is the isothermal-isobaric replica-exchange method proposed by Okabe et al. [20]. They demonstrated the effectiveness of this approach for systems of Lennard–Jones particles using isothermal-isobaric replica-exchange MC simulations (REMC). The potential energy was able to reach the low-energy region in the crystal phase, whereas in conventional MC simulations the energy remained high for those states.

This method is widely applicable and has been used for a diverse range of system types, such as hard spherocylinders [21], small mercury clusters [22], Glutamine Amides [23], and water confined within nanotubes [24]. The isothermal-isobaric replica-exchange algorithm inhibits kinetic trapping problems and, as such, can be used to greatly reduce or even eliminate hysteresis.

There are also significant speed increases to be gained when using this method. For example, in the constant NVT MD simulations of soft-core Gay–Berne molecules performed by Berardi et al. [25], equilibrium states were attained approximately 20% faster when compared to standard MD simulations.

In the implementation of this replica-exchange method, non-interacting replicas are prepared, each with a different set of temperatures and pressures. All the replicas are simulated independently and at the same time, with attempts made to exchange replicas of different pressures and temperatures at set intervals. Eventually, multiple canonical distributions equivalent to the number of replicas are obtained.

Whereas local minima trapping, problems are often seen in the results produced by conventional methods; in the replica-exchange method, where replicas may have various pressure and temperature values, structural fluctuations are larger. This allows the system to find the veridical minimum free-energy condition. It should be noted that two replicas can be switched only when their energy histograms overlap each other. Thus, many replicas are necessary near the phase transition regions. This is especially true for first-order phase transitions.

One tool that is particularly useful in the analysis of phase transitions is the Weighted Histogram Analysis Method (WHAM) [26]. This provides free-energy estimates with minimal statistical errors. It can also be used to calculate free-energy differences between phases at points where their probability distributions overlap.

The original WHAM method, as proposed by Kumar and co-workers [26], is an extension of prior work by Ferrenberg and Swendsen [27,28]. This method has been extensively used for the study of phase transitions and has seen use with systems of Lennard-Jones molecules [29], Cylinder Amyloids [30], lattice polymers [31] and various Mie-potential molecular models [32]. As such it is suitable for analysis of first-order phase transitions and the results produced using replica-exchange MC.

With the aforementioned points in mind, our objective in this study is to investigate the phase transitions of a rod-like Hess–Su liquid-crystal fluid. To this end, we shall employ the replica-exchange MC method, the results of which we shall compare with those obtained using the conventional MC method. Further to this, the WHAM will be an essential component of our analysis as it will allow us to visualize the free-energy surfaces for each temperature and pressure.

The following sections of this paper are arranged as follows: in Section 2, we give details of the molecular model used, the replica-exchange method, methods for calculating the heat capacity and bond order parameters, the weighted histogram analysis method, and the simulation conditions used in this work; Section 3 contains the results from these numerical simulations; concluding remarks are made in Section 4.

## 2. Method

### 2.1. Hess–Su Model

In this work, we perform MC simulations in the isobaric-isothermal ensemble (NPT), following the method used by Cuetos et al. [17]. The uniaxial and anisotropic liquid-crystal molecules simulated here are represented by the model of Hess–Su, as implemented by Steuer et al. [18]. The interaction energy between a pair of two such molecules is described by:

(1) $$\Phi \left(r,u_{1},u_{2}\right)=4\varepsilon \left[{\left(\frac{\sigma }{r}\right)}^{12}-{\left(\frac{\sigma }{r}\right)}^{6}\left\{1+\Psi \left(\hat{r},u_{1},u_{2}\right)\right\}\right],$$

where ε and σ are the usual Lennard–Jones dispersion and distance parameters, respectively, with σ being used as our unit of distance throughout this paper. $\hat{r}$ is the unit vector of r, the center to center vector between each molecule: $r=r_{2}-r_{1}$. The longest axes of the two molecules in the interacting pair are denoted by the orientation vectors $u_{1}$ and $u_{2}$. The anisotropy coefficients are $\varepsilon_{1}$ and $\varepsilon_{2}$, which define the effective shape of this molecular model. Specifically, the molecule behaves in a disk-like manner when $\varepsilon_{2}$ is positive and is rod-like when $\varepsilon_{2}$ is negative. Finally, the attractive term is written as:

(2) $$\Psi \left(\hat{r},u_{1},u_{2}\right)=5\varepsilon_{1}P_{2}\left(u_{1}\cdot u_{2}\right)+5\varepsilon_{2}\left[P_{2}\left(u_{1}\cdot \hat{r}\right)+P_{2}\left(u_{2}\cdot \hat{r}\right)\right]\;,$$

where $P_{2}\left(x\right)=\left(3x^{2}-1\right)/2$, which is the second Legendre Polynomial.

### 2.2. Simulation Conditions

Following the work of Steuer et al. [18,19], the anisotropy coefficients are chosen to be $\varepsilon_{1}=0.04$ and $\varepsilon_{2}=-0.08$ and the cut-off distance is set to $r_{c}=3.0\sigma$. Temperature, pressure, energy, internal energy, volume, and number density are expressed as non-dimensional values [33], where $T^{*}=k_{B}T/\varepsilon$, $P^{*}=P\sigma^{3}/\varepsilon$, $E^{*}=E/\varepsilon$, $U^{*}=U/\varepsilon$, $V^{*}=V/\sigma^{3}$ and $\rho^{*}=N/V^{*}$.

As our primary interest in this study is to examine the isotropic-nematic and nematic-solid phase transitions, it is essential that we be able to efficiently simulate high-density phases. This rules out using the Grand Canonical ensemble, as particle insertion methods are notoriously inefficient at high densities. Instead, the natural choice here is to perform our simulations in the isobaric-isothermal, constant NPT, ensemble. We choose to perform the simulations in this work with the smallest possible system size, given our choice of $r_{c}=3.0\sigma$ and the densities required to simulate nematic and solid phases. This permits us to simulate systems with N=256 molecules. The reasoning behind having such a low number of molecules is because replica-exchange transition probabilities are higher for larger systems.

### 2.3. Replica-Exchange Method

The implementation of the replica-exchange method used in this work is that of Okabe and co-workers [20]. We chose this method because it allows us to simulate individual systems in the constant isobaric-isothermal ensemble while permitting temperature and pressure exchanges to occur between systems at regular intervals of 7000 to 8000 MC cycles.

These exchanges are attempted between systems of adjacent temperatures and pressures with acceptance probabilities between systems m and n given by:

(3) $$\begin{array}{c} W\left(X,\beta_{m},P_{m}^{*}|X^{\prime },\beta_{n},P_{n}^{*}\right)=\left\{\begin{array}{cc} 1 & \mathrm{for}\;\Delta \leq 0\\ \exp(-\Delta ) & \mathrm{for}\;\Delta >0\;, \end{array}\right. \end{array}$$

where Δ is defined as:

(4) $$\Delta =\left(\beta_{m}-\beta_{n}\right)\left(U_{n}^{*}-U_{m}^{*}\right)+\left(\beta_{m}P_{m}^{*}-\beta_{n}P_{n}^{*}\right)\left(V_{n}^{*}-V_{m}^{*}\right)\;.$$

Here, $\beta_{\left(m,n\right)}$, $V_{\left(m,n\right)}^{*}$, $P_{\left(m,n\right)}^{*}$, and $U_{\left(m,n\right)}^{*}$ correspond to the inverse temperature, volume, pressure, and internal energy of the mth and nth replicas, respectively. In this paper, we prepare 572 replicas of the 256-particle system with pressures of $P^{*}=0.1$ to $P^{*}=7.2$ at intervals of $P^{*}=0.1$ and temperatures of $T^{*}=0.98$ to $T^{*}=1.05$ at intervals of $T^{*}=0.01$. The initial systems are all low-density isotropic states with near-zero nematic-order parameters. The replicas are exchanged in accordance with the Metropolis criterion written above in Equation (3).

We impose a restriction on our use of this equation such that attempted exchange moves are made between systems of equal temperature or of equal pressures, but never both at the same time. This was done in part to simplify the implementation of the replica-exchange method and because a more complicated exchange branch for both different temperatures and different pressures in a single exchange move would not yield appreciably different results.

Equilibrium states were obtained after 100,000,000 MC cycles, taking care to run for longer than the 10,000 cycles used in the prior study of Steuer et al. 2004 [19]. The results of these simulations are presented in the section that follows. We refer to these equilibrium states as the upward branch. In the second stage of this work, we perform identical simulations to those described above, differing only in that the initial system used for all replicas is a high-density solid state taken from the final configuration at $T^{*}=0.98$ and $P^{*}=7.2$. The results from these simulations will comprise the downward branch of our study of the hysteresis of this system.

In order to validate our simulation approach compared to conventional methods, we concurrently simulated the above two branches using the traditional MC method, without replica exchanges. This will allow a clear comparison between the two methods and demonstrate the advantages of the replica-exchange method.

### 2.4. Heat Capacity

One of benefits of recording the instantaneous energies and densities in our simulations is that it allows us to straightforwardly calculate the dimensionless molar heat capacity $c_{p}^{*}$. This is obtained using a similar relation to that previously used by Kronome et al. [34] for a Lennard-Jones based system:

(5) $$c_{p}^{*}=N\frac{<h^{*2}>-<h^{*}{>}^{2}}{T^{*2}},$$

where the non-dimensional molar enthalpy is given by $h^{*}=\left(E^{*}+P^{*}V^{*}\right)/N$.

The results from this analysis and the preceding simulation are presented in the following section.

### 2.5. WHAM

In the analysis of our simulation results, it is helpful to make use of the WHAM. This will aid us in identifying the precise locations of the phase transition points. To do this we require the density of states $n(U^{*},V^{*})$. This can be obtained using the method of Okumura et al. [29,35].

The molar free-energy surface $g^{*}$ can then be obtained as:

(6) $$g^{*}=-\ln\left(c_{0}n\left(U^{*},V^{*}\right)\exp\left(\frac{U^{*}}{N}+\frac{P^{*}}{\rho^{*}}\right)\right),$$

where $c_{0}$ is a normalisation constant [36] which does not affect the relative value of $g^{*}$. In this work we set it to unity.

### 2.6. Bond Order Parameters

In this study, because we are also going to examine the high-density region of the phase diagram it is useful to characterise the type of solid phase that may be present. Here we use the order parameter developed by Halperin and Nelson [37]. Bond-order parameters are often used to distinguish between liquid and possible solid structures. This function is defined as:

(7) $$B_{\nu }=\frac{1}{\nu N}∥\sum_{j=1}^{N}\sum_{k=1}^{\nu }\exp\left(i\nu \phi_{jk}\right)∥,$$

where $\phi_{jk}$ is the angle between the bond which connects particle *j* and *k* and a fixed reference frame. A bond is a straight line which links the centres of mass of two neighboring particles. *N* is the number of particles in the system. ν is the number of nearest-neighbor bonds.

When examining liquid–solid transitions, ν=4 and ν=6 are most commonly chosen as they are useful to identify BCC (body-centered cubic), FCC (face-centered cubic) and HCP (hexagonal close-packed) structures.

## 3. Results

### 3.1. Conventional MC

To validate our simulation set up and set a baseline from which to measure the precise phase transition points in this system, we first perform a series of conventional constant NPT MC simulations. These are identical in all respects to the replica-exchange method, as described in the previous section, apart from one point which is that we do not attempt to perform replica-exchange moves at any stage during the simulation.

For these simulations, two branches of the phase diagram as investigated so as to measure the degree of hysteresis in this system. The first stage required us to start from a low-density isotropic phase with gradually increasing pressure at a fixed temperature of $T^{*}=1.0$. After we obtain a high-density equilibrium state at $P^{*}=5.7$, this is used as the initial configuration for the downward branch, as described in the previous section.

**Figure 1:**
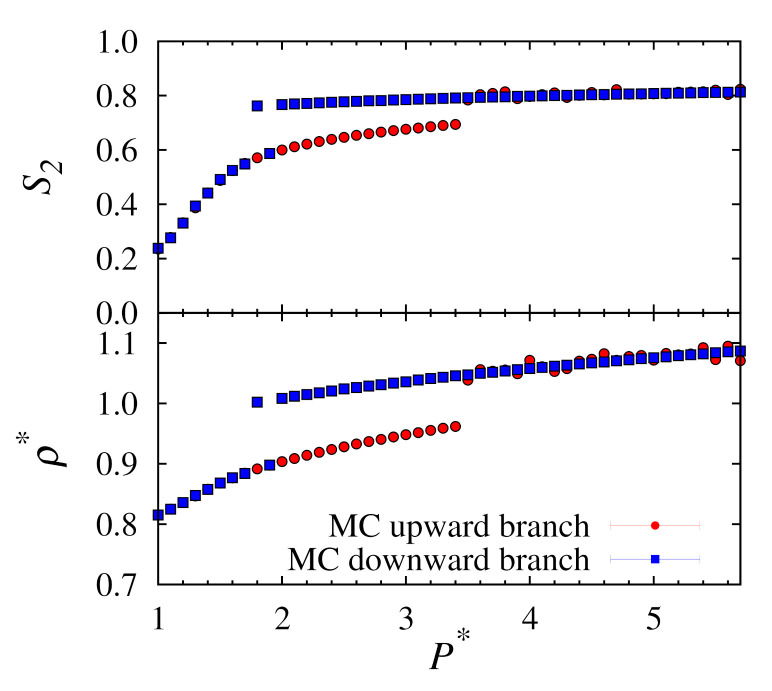
Plots of nematic order $S_{2}$ (**top**) and number density $\rho^{*}$ (**bottom**) versus pressure $P^{*}$ for upward and downward branches using conventional MC at $T^{*}=1.0$.

The results obtained from these simulations were taken as the average values of density and nematic order over the final 20,000,000 MC cycles from simulations run over a total of 200,000,000 MC cycles from their initial systems. Each simulation was performed using a single CPU core from a conventional Intel Xeon processor. Nematic order $S_{2}$ is calculated using the standard second Legendre polynomial function. A plot of results is given in Figure 1.

It is apparent that for the downward branch that the transition from solid to nematic phases occurs inconsistently with pressure. For example, we see a nematic phase for $P^{*}=1.9$ but the point at $P^{*}=1.8$ remains in the solid phase. It should be expected that after a large number of cycles this point would eventually also transition to the nematic phase. However, this is a stochastic process and we have no sure way of knowing when this might occur. In addition, for the upward branch, the equilibrium densities are not uniform at higher pressures. It is likely that some of these states are trapped in a metastable solid phase. These situations clearly demonstrate the limitations of the conventional MC approach.

### 3.2. Replica-Exchange MC

**Figure 2:**
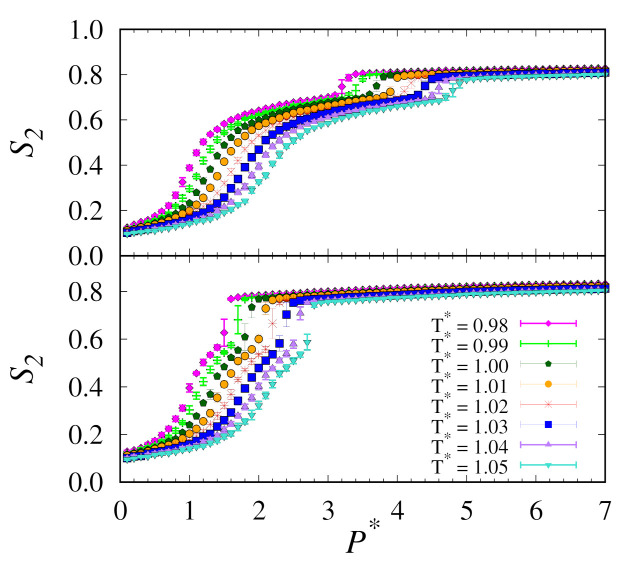
Plots of nematic order $S_{2}$ versus pressure $P^{*}$ for upward (**top**) and downward (**bottom**) branches using replica-exchange MC at temperatures ($T^{*}$) as indicated by the legend.

Moving on to the replica-exchange method, these simulations were performed over 100,000,000 MC cycles. These are half as many as our conventional MC simulations. Here, we used a supercomputer with 576 cores at a rate of approximately 8,000,000 MC cycles per day. Results from both the upward and downward branches of our replica-exchange MC simulations for all temperature and pressure intervals studied are shown in Figure 2.

**Figure 3:**
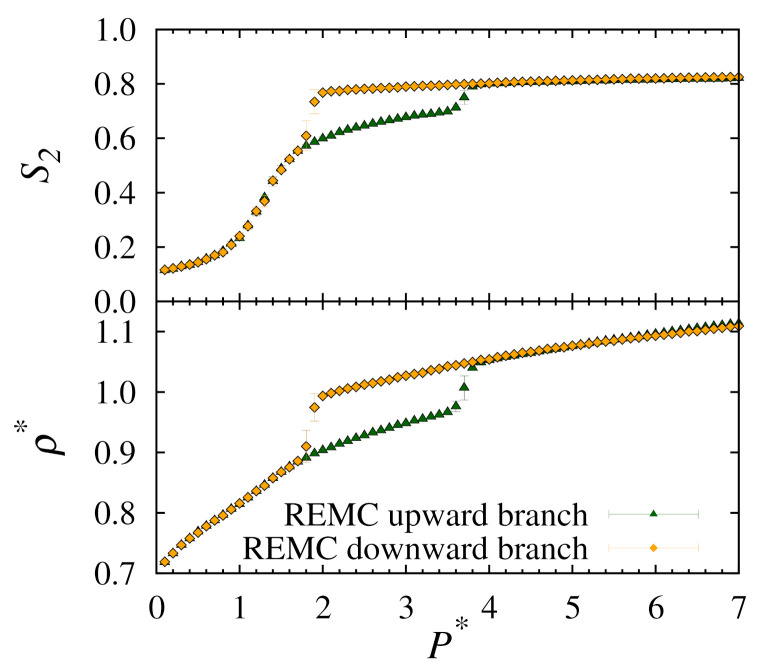
Plots of nematic order $S_{2}$ (**top**) and number density $\rho^{*}$ (**bottom**) versus pressure $P^{*}$ for upward and downward branches using replica-exchange MC at $T^{*}=1.0$.

In order to compare more directly with our previous conventional MC results, in Figure 3 we present a plot of the density and nematic ordering for $T^{*}=1.0$ only.

Clearly, our results show that, when compared to conventional MC simulations, for half the number of cycles the replica-exchange method is able to predict consistent solid-nematic phase transition points and has uniform density for the solid phases at higher pressures.

**Figure 4:**
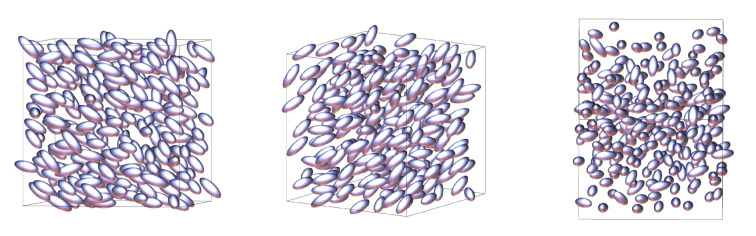
Snapshot of a nematic phase at $T^{*}=1.0$ and $P^{*}=3.0$ from three different angles.

**Figure 5:**
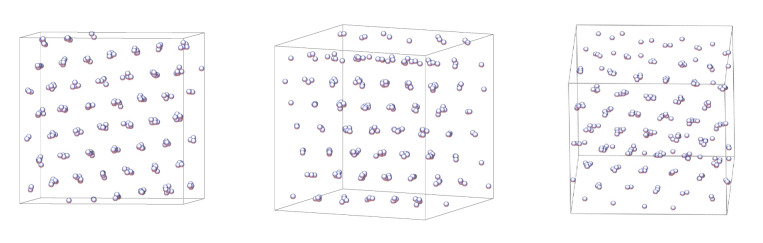
Snapshot of a solid phase at $T^{*}=1.0$ and $P^{*}=7.0$ from three different angles. To help with the visualisation of the structure of this phase, all molecules have been rendered as small spherically symmetric spheres.

It is evident from the plots of nematic order and density for this system that at $T^{*}=1.0$ and $P^{*}=3.7$ there is a transition from the nematic phase to another more ordered, higher density, solid phase. We present our nematic phase as shown in Figure 4. Typically for liquid-crystals fluids, this would be the smectic-A phase. However, upon closer inspection, we see that for the Hess–Su model studied here this phase transition instead goes directly to a crystalline solid phase, as shown in Figure 5.

This behaviour was also reported in a prior simulation study by Steuer et al. [18], who observed that for $\varepsilon_{1}=0.04$ and $\varepsilon_{2}=-0.08$ there is no stable smectic phase. It was conjectured, however, that different parameter values may yield stable smectic phases and this would be an interesting subject for possible future research in this area.

### 3.3. Heat Capacity

**Figure 6:**
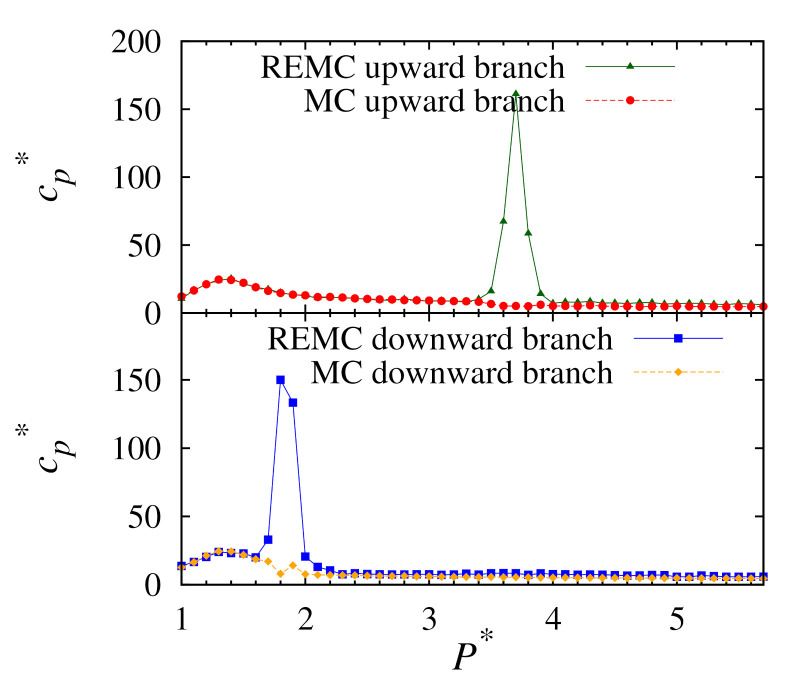
Plots of heat capacity ${c_{p}}^{*}$ versus pressure $P^{*}$ for upward (**top**) and downward (**bottom**) branches using conventional MC and replica-exchange MC at $T^{*}=1.0$.

To examine the phase transitions, we look for peaks in the heat capacity profiles. These are calculated using Equation (5). Profiles from the simulations performed in this work are presented in Figure 6. For the replica-exchange method, it is evident that there are two-phase transition points: a broad peak and another much sharper peak. These correspond to a second-order isotropic-nematic phase transition and a first-order nematic-solid phase transition, respectively. In all cases, the upward and downward branches give an isotropic-nematic phase transition point at $P^{*}=1.3$ for a temperature of $T^{*}=1.0$. It is expected that both methods would give the same result here as second-order transitions are not normally problematic for conventional MC. However, the location of the nematic-solid phase transition point varies according to the method used and if the simulation was performed on the upward or downward branches. In our case, it was found that neither branch of the conventional MC simulations produced peaks for the nematic-solid transitions. However, the replica-exchange method gives a pressure of $P^{*}=3.7$ for the upward branch. Likewise, for the downward branch the replica-exchange method gives the transition at $P^{*}=1.8$.

**Figure 7:**
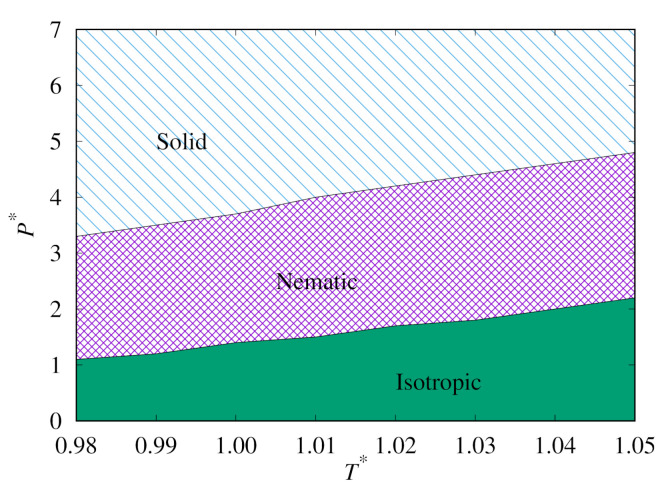

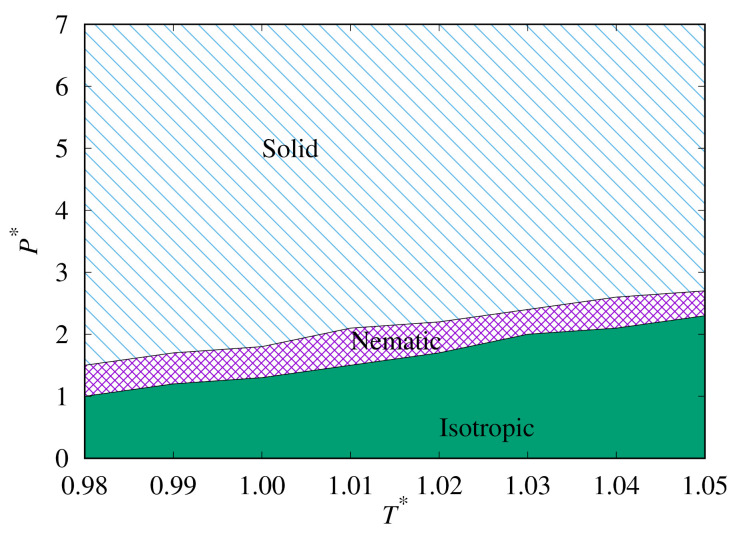
Phase diagram plots for phase transition lines predicted from replica-exchange MC for the upward (**top**) and downward (**bottom**) branches.

Next, we plot the heat capacity peaks for both our replica-exchange MC simulations, as shown in Figure 7. This clearly shows the phase behaviour of the Hess–Su model for temperatures close to $T^{*}=1.0$. It also demonstrates the extent of the hysteresis in this system.

### 3.4. Overlapping Energy Distributions

**Figure 8:**
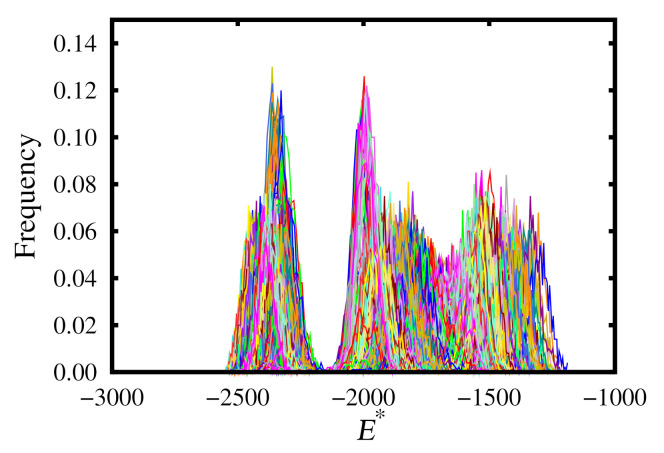
Replica-exchange energy histograms for the upward branch.

The replica-exchange method works due to the increased probability of phase transitions occurring during the simulations. This is due to overlapping free-energy distributions at particular temperatures and pressures for the different phases. An example of this from our simulations may be found in Figure 8. Note that it is important to select temperature and pressure intervals such that these histograms overlap.

### 3.5. WHAM Landscape Pathways

**Figure 9:**
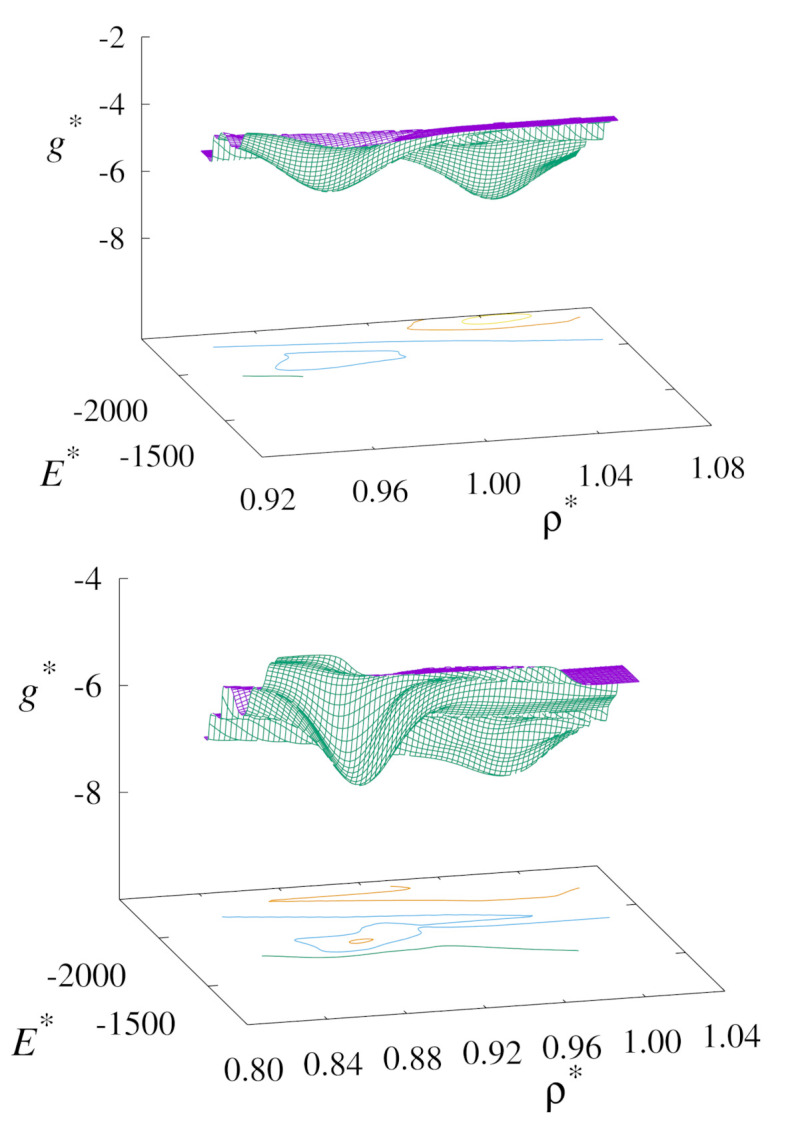
Plots of the free-energy surfaces for the nematic-solid phase transition for the upward (**top**) and downward (**bottom**) branches using replica-exchange MC at $T^{*}=1.0$.

Taking this analysis further, we make a detailed examination of the phase transition points using the WHAM approach. In Figure 9 we show the free-energy landscape of the nematic to solid phase transition in our replica-exchange simulations. This method serves to highlight the locations of the phase transitions points from our simulations. These are the locations where we observed phase coexistence, which is characterised by the presence of double minima in the free-energy landscapes.

### 3.6. Bond-Order Parameters

**Figure 10:**
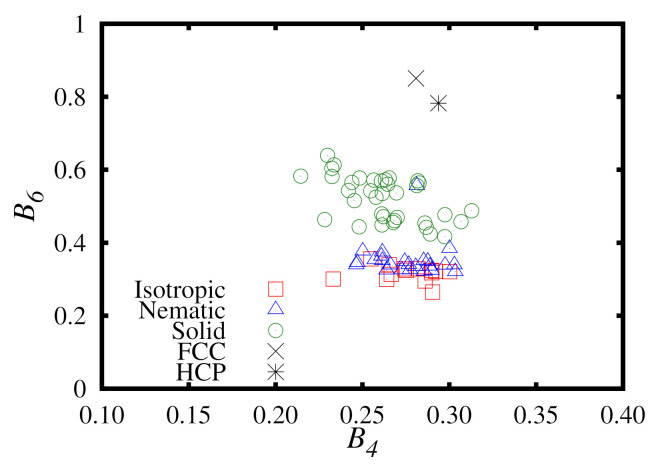
A plot of $B_{4}$ versus $B_{6}$ for the Hess–Su liquid-crystal model studied in this work using the upward branch of the replica-exchange method simulations. The phase of each point is denoted in the legend. For comparison, data points for perfect FCC and HCP crystals are also included.

We compare the local bond order parameters, $B_{4}$ and $B_{6}$, of the different phases simulated in this work. These results are presented in Figure 10. It is clear that there is no appreciable increase in $B_{4}$ from the isotropic to the solid phase. However, we see a pronounced jump in $B_{6}$, which corresponds to an HCP structure. This identification of an HCP structure is in an agreement with the system snapshots observed in Figure 5, from which we were able to observe hexagonal ordering in multiple axes or orientation. Despite extensive investigation, no FCC order was observed in any of the phases from our simulations.

## 4. Conclusions

In this paper, we have performed temperature and pressure replica-exchange MC simulations of a simple model liquid-crystal fluid. In agreement with prior work by Steuer et al. [18], we report a second-order isotropic-nematic phase transition and the complete absence of a stable smectic phase for the range of temperatures and pressures investigated. Instead, we found a direct nematic to solid phase transition which is strongly first order. Importantly, we found the nematic phase is stable only over a narrow range of densities. For example, any nematic state at $T^{*}=1.0$ with a number density above $\rho^{*}=0.98$ is almost certainly meta-stable. Therefore, care must be taken when using this potential at intermediate-to-high pressures. It should be noted of course that the presence of stable phases and their phase transitions is heavily dependent on the particular molecular characteristics selected for the study [38].

The results showed how the replica-exchange method enables us to explore a broad range of phase space, which effectively samples the equilibrium, and provided an efficient way of accessing wider free-energy regions. We have established that the replica-exchange method can be used to find heat capacity peaks for the Hess–Su model of liquid crystals, and obtained evidence that it can find points of phase co-existence. This approach is also more computationally efficient for non-spherically symmetric molecules when compared to the traditional MC method. This demonstrates that this algorithm is of great use for the study of complex materials, and it should be emphasized that it should play an integral role in many future simulation studies.

## Data Availability

Not applicable.

## Abbreviations

The following abbreviations are used in this manuscript:

MC	Monte Carlo
REMC	Replica-Exchange Monte Carlo
WHAM	Weighted Histogram Analysis Method

## References

[1] Chodera J.D., Swope W.C., Pitera J.W., Seok C., Dill K.A. (2007). Use of the weighted histogram analysis method for the analysis of simulated and parallel tempering simulations. J. Chem. Theory Comput..

[2] Andrienko D. (2018). Introduction to liquid crystals. J. Mol. Liq..

[3] Singh S. (2000). Phase transitions in liquid crystals. Phys. Rep..

[4] Luckhurst G., Stephens R., Phippen R. (1990). Computer simulation studies of anisotropic systems. XIX. Mesophases formed by the Gay-Berne model mesogen. Liq. Cryst..

[5] Frenkel D., Lekkerkerker H., Stroobants A. (1988). Thermodynamic stability of a smectic phase in a system of hard rods. Nature.

[6] Frenkel D., Mulder B. (1985). The hard ellipsoid-of-revolution fluid: I. Monte Carlo simulations. Mol. Phys..

[7] Hess S., Su B. (1999). Pressure and isotropic-nematic transition temperature of model liquid crystals. Z. Naturforsch. A.

[8] Greschek M., Schoen M. (2011). Finite-size scaling analysis of isotropic-nematic phase transitions in an anisometric Lennard-Jones fluid. Phys. Rev. E.

[9] Giura S., Schoen M. (2014). Density-functional theory and Monte Carlo simulations of the phase behavior of a simple model liquid crystal. Phys. Rev. E.

[10] Greschek M., Schoen M. (2011). Orientational prewetting of planar solid substrates by a model liquid crystal. J. Chem. Phys..

[11] Greschek M., Melle M., Schoen M. (2010). Isotropic–nematic phase transitions in confined mesogenic fluids. The role of substrate anchoring. Soft Matter.

[12] Melle M., Schlotthauer S., Mazza M.G., Klapp S.H., Schoen M. (2012). Defect topologies in a nematic liquid crystal near a patchy colloid. J. Chem. Phys..

[13] Stieger T., Schoen M., Mazza M.G. (2014). Effects of flow on topological defects in a nematic liquid crystal near a colloid. J. Chem. Phys..

[14] Berardi R., Emerson A.P.J., Zannoni C. (1993). Monte Carlo investigations of a Gay—Berne liquid crystal. J. Chem. Soc. Faraday Trans..

[15] De Miguel E., Rull L.F., Chalam M.K., Gubbins K.E. (1991). Liquid crystal phase diagram of the Gay-Berne fluid. Mol. Phys..

[16] Konishi Y., Tokoro H., Nishino M., Miyashita S. (2008). Monte Carlo simulation of pressure-induced phase transitions in spin-crossover materials. Phys. Rev. Lett..

[17] Cuetos A., Martınez-Haya B., Rull L., Lago S. (2002). Monte Carlo study of liquid crystal phases of hard and soft spherocylinders. J. Chem. Phys..

[18] Steuer H., Hess S., Schoen M. (2003). Pressure, alignment and phase behavior of a simple model liquid crystal. A Monte Carlo simulation study. Physica A.

[19] Steuer H., Hess S., Schoen M. (2004). Phase behavior of liquid crystals confined by smooth walls. Phys. Rev. E.

[20] Okabe T., Kawata M., Okamoto Y., Mikami M. (2001). Replica-exchange Monte Carlo method for the isobaric–isothermal ensemble. Chem. Phys. Lett..

[21] Basurto E., Gurin P., Varga S., Odriozola G. (2020). Ordering, clustering, and wetting of hard rods in extreme confinement. Phys. Rev. Res..

[22] Šarmanová M., Vítek A., Ćosić R., Kalus R. (2019). Photoabsorption markers of pressure-induced phase changes in small mercury clusters. A case study on Hg 8. RSC Adv..

[23] Ganguly A., Thiel W., Crane B.R. (2017). Glutamine amide flip elicits long distance allosteric responses in the LOV protein Vivid. J. Am. Chem. Soc..

[24] Nomura K., Kaneko T., Bai J., Francisco J.S., Yasuoka K., Zeng X.C. (2017). Evidence of low-density and high-density liquid phases and isochore end point for water confined to carbon nanotube. Proc. Natl. Acad. Sci. USA.

[25] Berardi R., Zannoni C., Lintuvuori J.S., Wilson M.R. (2009). A soft-core Gay–Berne model for the simulation of liquid crystals by Hamiltonian replica exchange. J. Chem. Phys..

[26] Kumar S., Rosenberg J.M., Bouzida D., Swendsen R.H., Kollman P.A. (1992). The weighted histogram analysis method for free-energy calculations on biomolecules. I. The method. J. Comput. Chem..

[27] Ferrenberg A.M., Swendsen R.H. (1988). New Monte Carlo technique for studying phase transitions. Phys. Rev. Lett..

[28] Ferrenberg A.M., Swendsen R.H. (1989). Optimized monte carlo data analysis. Comput. Phys..

[29] Okumura H., Okamoto Y. (2004). Molecular dynamics simulations in the multibaric–multithermal ensemble. Chem. Phys. Lett..

[30] Zhang H., Xi W., Hansmann U.H., Wei Y. (2017). Fibril–barrel transitions in cylindrin amyloids. J. Chem. Theory Comput..

[31] Panagiotopoulos A.Z., Wong V., Floriano M.A. (1998). Phase equilibria of lattice polymers from histogram reweighting Monte Carlo simulations. Macromolecules.

[32] Weidler D., Gross J. (2016). Transferable anisotropic united-atom force field based on the Mie potential for phase equilibria: Aldehydes, ketones, and small cyclic alkanes. Ind. Eng. Chem. Res..

[33] Allen M.P., Tildesley D.J. (2017). Computer Simulation of Liquids.

[34] Kronome G., Kristóf T., Liszi J., Szalai I. (1999). Heat capacities of two-centre Lennard–Jones fluids from NpT ensemble Monte Carlo simulations. Fluid Phase Equilib..

[35] Okumura H., Okamoto Y. (2006). Multibaric–multithermal ensemble molecular dynamics simulations. J. Comput. Chem..

[36] Kaneko T., Bai J., Yasuoka K., Mitsutake A., Zeng X.C. (2014). Liquid-solid and solid-solid phase transition of monolayer water: High-density rhombic monolayer ice. J. Chem. Phys..

[37] Halperin B., Nelson D.R. (1978). Theory of two-dimensional melting. Phys. Rev. Lett..

[38] Kralj S., Cordoyiannis G., Jesenek D., Zidanšek A., Lahajnar G., Novak N., Amenitsch H., Kutnjak Z. (2012). Dimensional crossover and scaling behavior of a smectic liquid crystal confined to controlled-pore glass matrices. Soft Matter.

